# A Conservation-Based Approach to Compensation for Livestock Depredation: The Florida Panther Case Study

**DOI:** 10.1371/journal.pone.0139203

**Published:** 2015-09-30

**Authors:** Caitlin E. Jacobs, Martin B. Main

**Affiliations:** Department of Wildlife Ecology and Conservation, University of Florida Institute of Food and Agricultural Sciences, Gainesville, Florida, United States of America; University of Illinois at Urbana-Champaign, UNITED STATES

## Abstract

Calf (*Bos taurus*) depredation by the federally endangered Florida panther (*Puma concolor coryi*) on ranches in southwest Florida is an important issue because ranches represent mixed landscapes that provide habitat critical to panther recovery. The objectives of this study were to (1) quantify calf depredation by panthers on two ranches in southwest Florida, and (2) develop a habitat suitability model to evaluate the quality of panther hunting habitat on ranchlands, assess whether the model could predict predation risk to calves, and discuss its potential to be incorporated into an incentive-based compensation program. We ear-tagged 409 calves with VHF transmitters on two ranches during 2011–2013 to document calf mortality. We developed a model to evaluate the quality of panther hunting habitat on private lands in southwest Florida using environmental variables obtained from the Florida Natural Areas Inventory (FNAI) Cooperative Landcover Database and nocturnal GPS locations of panthers provided by the Florida Fish and Wildlife Conservation Commission (FWC). We then tested whether the model could predict the location of calf depredation sites. Tagged calf loss to panthers varied between the two ranches (0.5%/yr to 5.3%/yr) and may have been influenced by the amount of panther hunting habitat on each ranch as the ranch that experienced higher depredation rates contained a significantly higher probability of panther presence. Depredation sites of tagged calves had a significantly greater probability of panther presence than depredation sites of untagged calves that were found by ranchers in open pastures. This suggests that there may be more calves killed in high risk environments than are being found and reported by ranchers and that panthers can hunt effectively in open environments. It also suggests that the model may provide a means for evaluating the quality of panther hunting habitat and the corresponding risk of depredation to livestock across the landscape. We suggest that our approach could be applied to prioritize and categorize private lands for participation in a Payment for Ecosystem Services program that compensates landowners for livestock loss and incentivizes conserving high quality habitat for large carnivores where livestock depredation is a concern.

## Introduction

Conflict between large carnivores and humans is a global issue that has become an important aspect of large carnivore conservation. Livestock depredation is often the principal reason for this conflict and can lead farmers to kill predators in retaliation or as a preventative measure [1]. As a result, livestock depredation is considered one of the driving forces behind the worldwide decline of large carnivores [1,2,3]. While compensation programs are often established to address the issue of livestock depredation, some of the most common programs are highly criticized due to problems such as moral hazard [4,5], high transaction costs [6], unconfirmed losses, and the difficultly of finding depredations [5,7]. It is therefore important to consider new approaches to compensation programs in order to conserve and, where needed, recover large carnivore species.

Once distributed throughout the southeastern United States, the federally endangered Florida panther (*P*. *concolor coryi*) now occupies less than 5% of its historic range due to habitat loss, prey declines, and attempts to eradicate panthers in the early 1900s [8]. The only breeding population of Florida panthers occurs in south Florida and during the 1980s the panther population was thought to be as low as 20–30 adults, but due to protection under the Endangered Species Act and recent recovery efforts the population is now believed to range from 100–180 panthers of breeding age [9,10]. This population growth has led to an increase in panther use of private ranchlands, resulting in an increase of verified calf (*B*. *taurus*) depredations by panthers in south Florida [10], which has raised concerns and requests for compensation (L.Priddy, Rancher and Commissioner, Florida Fish and Wildlife Conservation Commission (FWC), personal communication).

**Fig 1:**
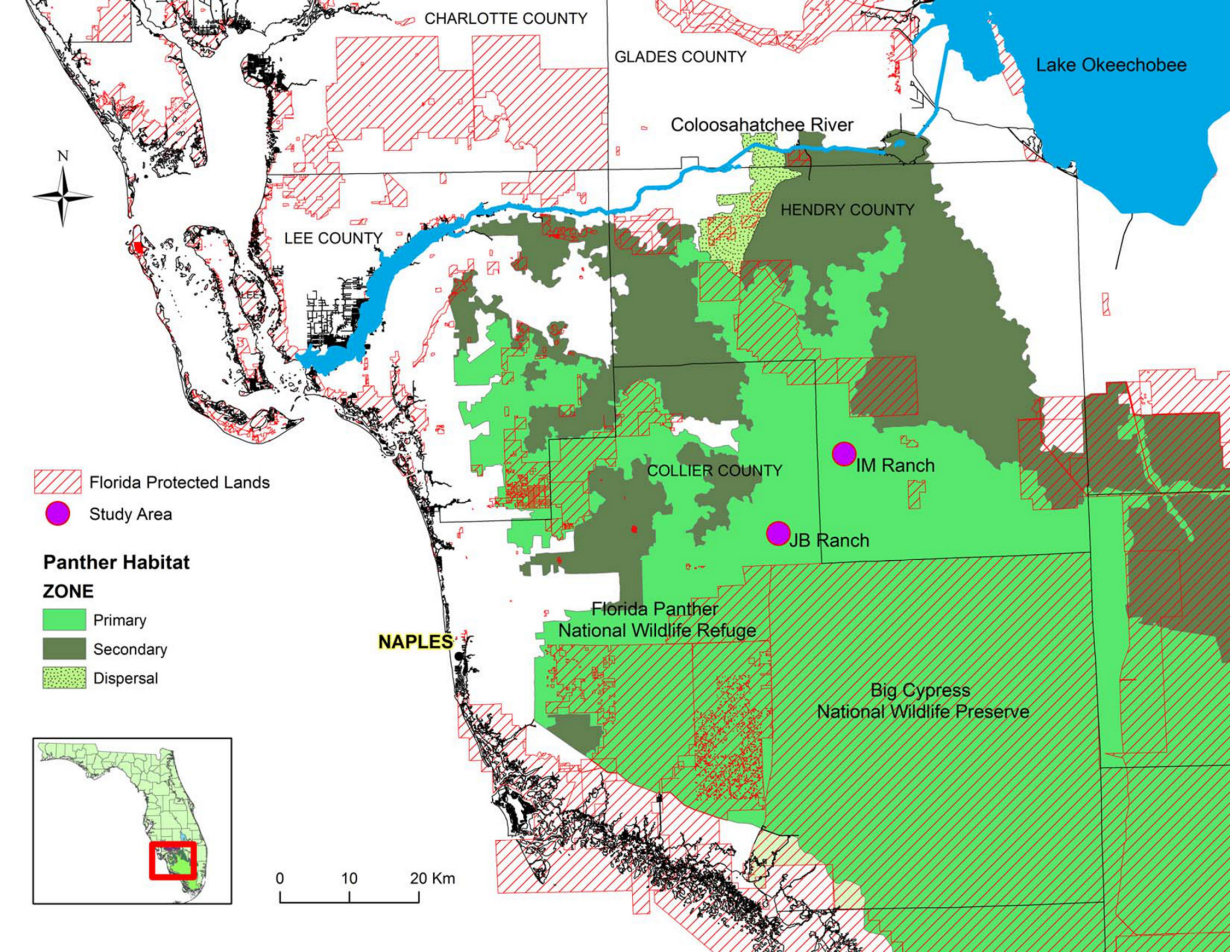
Location of the JB Ranch (JB) and Immokalee Ranch (IM) study areas in the Primary Zone (high quality panther habitat) in southwest Florida.

Florida panther recovery efforts have included the designation of primary, secondary, and dispersal panther habitat zones in south Florida (Fig 1). Panther habitat zones represent both public and private lands identified as essential to the long-term survival of the Florida panther [8]. Briefly described, the primary zone includes all lands essential for the survival of the Florida panther in the wild, the secondary zone includes lands which panthers may currently use that are contiguous with the primary zone and where expansion of the Florida panther population is most likely to occur, and the dispersal zone was identified as an area needed for panthers to disperse north of the Caloosahatchee River [8]. Private lands encompass approximately 27% of the primary zone, which supports the core range of the Florida panther, 60% of the secondary zone, and nearly the entire dispersal zone [11]. Many private lands contain cattle ranches, which are low-intensity land use operations that typically support a mosaic of different natural land cover types used by the Florida panther and its prey [12, 13,14,15].

Private lands play a critical role in providing habitat that is needed for panther conservation and recovery. Addressing the issue of calf depredation is important, both to ranchers and to the successful recovery of Florida panthers because recovery efforts would suffer if calf depredation dissuaded ranchers from maintaining natural areas or resulted in land conversion to higher intensity agricultural production or urban/suburban development. To understand the impact of panthers on the ranching industry and to help inform potential compensation strategies, the objectives of this study were to (1) quantify calf depredation by the Florida panther on two ranches in southwest Florida, and (2) develop a habitat suitability model to evaluate the quality of panther hunting habitat on ranchlands, assess whether the model could predict predation risk, and discuss its potential to be incorporated into an incentive-based compensation program.

## Materials and Methods

### Study Area

Southwest Florida has a subtropical climate with a cooler dry season from October to May and a very warm, rainy season from June to September [16]. The region is comprised of a diverse landscape that includes large tracts of state and federal conservation lands as well as privately owned lands. State and federal lands are largely comprised of forests and freshwater wetlands, while private lands contain these natural areas in addition to agricultural crops, citrus groves, and cattle pastures.

We monitored calves on two large commercial beef ranches, the JB Ranch (JB) and the Immokalee Ranch (IM), both of which are located within the primary zone of panther habitat in southwest Florida (Fig 1). Both study ranches are primarily engaged in raising cattle and support a mosaic of land cover types including improved and unimproved pastures, hardwood hammocks, and wetlands. On both ranches, female cattle are pregnancy tested and then put out to graze over large pastures where they give birth and raise their calves. Neither ranch moved calves to more secure pastures or into protective fencing enclosures to reduce predation risk.

The JB Ranch contains 3,013 ha of cattle grazing land (R. Priddy, JB Ranch, personal communication) and the IM Ranch contains 20,639 ha of cattle grazing land (C.W. Stoner, IM Ranch, personal communication). Study cattle herds (*n* = ~100/ranch/yr) were associated with specific study areas on each ranch and these differed slightly in size (JB: 268 ha; IM: 913 ha), but did not differ in cattle density (JB: 0.37 cow-calf pairs/ha; IM: 0.38 cow-calf pairs/ha). White-tailed deer (*Odocoileus virginianus*), feral hogs (*Sus scrofa*), and other prey species of the Florida panther occurred on the ranches, as well as other potential calf predator species such as black bears (*Ursus americanus*), coyotes (*Canis latrans*), bobcats (*Lynx rufus*) and alligators (*Alligator mississippiensis*). Black vultures (*Coragyps atratus*) and turkey vultures (*Cathartes aura*), which are reported to contribute to calf loss, were found on both ranches as well.

### Ethical Statement

This study was carried out in strict accordance with guidelines as established for ethical research involving animals by the University of Florida Institute for Food and Agricultural Sciences Animal Research Assurance Committee (ARAC; Protocol Approval Number: 001–11IMM). The UF/IFAS ARAC reviews all methodologies and was the designated review and approval authority for UF/IFAS research involving non-regulated animals (wildlife, fisheries, livestock) that fall outside the jurisdiction of the IACUC. Permission to conduct this research on private lands was provided by the owners of the JB and Immokalee Ranches.

### Calf Depredation

To quantify calf depredation we monitored 409 calves (*n* = ~100/ranch/yr) during September-April of 2011–12 and 2012–13 on the JB and IM ranches. Calves were captured by experienced cattlemen and ear-tagged with a 20 g VHF radio transmitter containing a 2-hr mortality switch using standard ear tag pliers (Advanced Telemetry Systems, Isanti, MN, USA). After tagging, the calves were immediately released and returned to the mother cow. We obtained visual records of each tagged calf every other day until calves were rounded up in March-April for marking and branding, during which time ear tags were removed.

The number and timing of calves tagged, the percentage and age structure of tagged calves in each study herd, and total monitoring effort differed between ranches due primarily to differences in study herd sizes. On JB Ranch, we tagged 100% of the calves in the study herd during each field season (n = 190 calves total). Calves were born and tagged over a period of approximately five months from September-February, which resulted in a study herd of tagged calves of different ages and weights as the field season progressed. Calves in the JB study herd were monitored for 31 weeks during September-April of 2011–12 and 2012–13. The IM Ranch study herd was larger and we tagged approximately 30% of the total number of calves present in the study herd during each field season (n = 219 calves total). All calves tagged on IM Ranch were born during November-December, which resulted in a study herd of tagged calves of similar ages and weights as the field season progressed. The IM study herd was monitored for 23 weeks and 16 weeks during November 2011-April 2012 and November 2012-March 2013, respectively.

We located dead calves using radio telemetry, and identified the predator responsible by evaluating cause of death (location of puncture wounds, bruising, lacerations), feeding patterns, species sign (tracks, scat), and site characteristics (location, environment, carcass cached). We recorded the age and estimated weight for each calf mortality and the FWC verified all panther depredations. We placed two trail cameras (Bushnell Trophy Cam, Bushnell Outdoor Products, Overland Park, KS, USA) at each depredation site to confirm the predator responsible and to evaluate the age and sex of panthers killing calves.

We calculated the proportion of tagged calves that were lost to panthers, other predators, and non-predation mortalities within each ranch. We used a Chi Square (SPSS 22, IBM, New York, NY, USA) to test for a difference in the proportion of mortalities between JB and IM to test the hypothesis that the ranch which provided the best panther hunting habitat would incur more depredations. We used 9 panther depredations on JB Ranch to test whether panthers selected for smaller calves and assumed that the age of the calf was correlated with the size of the calf. We compared the age of tagged calves killed by panthers with the mean age of all available tagged calves at the time of the depredation event using a Wilcoxon signed rank test (SPSS 22, IBM, New York, NY, USA) to determine whether calves killed by panthers were younger than the average age of all available calves. We performed a survival analysis to determine whether rates of calf mortality differed between study areas and field seasons. We included all mortality events of tagged calves in the analysis and used the known fate option in program MARK to analyze data [17]. Known fate models estimate the survival probability between two sampling occasions and are often used with radio-tagged individuals. Because the fate of each individual is recorded, known fate models do not have to model detection probability and the encounter probability is 1.0. This is in contrast to situations where the fate of marked individuals is unknown and the encounter probability is < 1.0 [17]. Individuals were “censored” (treated as an unknown fate in MARK) if their tag was known to have failed or fallen off, which occurred with only one calf. Three calves were abandoned by their mothers and these calves were counted as “removals” in MARK. We collapsed the daily monitoring effort into weekly periods and classified each individual as alive or dead for each week. We used the small sample correction of AIC to determine model rankings.

We also recorded depredations of untagged calves found during the study on both ranches, but percent losses of untagged calves could not be calculated because depredations may also have occurred among untagged calves that went undetected. The majority of untagged calves were found by investigating clusters of vultures or through identifying stressed mother cows that appeared to be searching for their calves. We recorded the location and estimated weight for untagged calf depredations.

### Panther Hunting Habitat Model and Predation Risk

We developed a panther hunting habitat model for all private lands within the panther primary, secondary, and dispersal zones [11]. We tested whether our model could predict areas of high predation risk to livestock by comparing the amount and quality of panther hunting habitat between study areas and by evaluating the ability of the model to identify documented calf depredation locations as areas of high predation risk.

To model panther hunting habitat we used the species distribution model MaxEnt v. 3.3.3k [18], which compares environmental variables at species location sites to the same variables at 10,000 random locations [19]. We chose MaxEnt over Resource Selection Function because while both models are mathematically similar, hold similar assumptions, and provide the probability of selection, MaxEnt is a faster model to develop, especially good at handling complex interactions between response and predictor variables, less sensitive to small sample sizes, contains tools for statistical interpretation, and provides ArcGIS compatible outputs [19,20,21].

We defined panther hunting habitat as the habitat used by panthers between 1900–0700 (nocturnal habitat use) because this is when panthers are the most active and the primary time when they hunt [12,22]. To identify panther hunting habitat we used location data recorded from 10 GPS-collared panthers (7 males, 3 females) during 2005–2009 [12]. These data were collected by the FWC using four different types of collars (Tellus, Telonics, Lotek, Tellus-GSM) with fix schedules ranging from 1 hr to 7 hrs. We selected a random subset of 100 nocturnal location records per individual panther to eliminate bias from panthers that had greater numbers of location records (S1 Fig, S1 Dataset). We excluded all points that occurred within the Okaloacoochee Slough State Forest and Corkscrew Regional Ecosystem Watershed (S1 Fig) because these conservation areas are dominated by wetlands and land cover differs substantially from cattle ranches and other privately-owned lands, most of which have greater amounts of upland environments and engage in some form of agricultural production. We selected the 10,000 random points from the same region as the sample data by constructing minimum convex polygons around nighttime GPS locations for each individual panther [23,24]. Finally, we projected the panther hunting habitat model onto our area of interest, i.e., private lands within the primary, secondary, and dispersal panther habitat zones in southwest Florida.

We used eight landscape variables in our hunting habitat model, two of which (distance from edge and land cover) had multiple subcategories (Table 1). All variables except cattle density were generated from the Cooperative Land Cover (CLC) v.2.3 FNAI database and were prepared as raster layers in ArcGIS using a cell size of 10 m. We reclassified the CLC land cover categories into similar land cover classes, designated each as either cover or open environments and forested or non-forested, and excluded all land cover classes that represented <1% of the study area (e.g., water bodies; S1 Table). The final land cover layer included eight classes (Table 1). We used a scale of 4.5 km^2^ (circular with *d* = 2.38 km) to calculate edge density, percent forest cover, and dominant land cover, because 4.5 km^2^ represents the average area used by an individual panther over a 24-hour period based on location data collected every hour from 13 GPS-collared Florida panthers (3 females, 10 males) during February 2005-December 2011 [25]. Cattle density data was generated by Robinson et al. [26] who used a combination of subnational and national cattle estimates as well as predictor variables (vegetation, climate, topography, and demography) to create regional cattle density estimates in a GIS format. All variables were assessed for correlation using principal components analysis and variables were retained in the model if correlations were <0.55 [27,28]. All variables were treated as continuous except for landcover and dominant landcover.

**Table 1:** Description of variables included in the panther hunting habitat model for private lands in the primary, secondary, and dispersal zones of panther habitat in southwest Florida.

Variable	Description	Hypothesis
Cattle Density	# of cattle/km^2^ [26].	Areas of low cattle density will contain a high probability of panther presence
Distance from edge (m)	Distance from edge between cover and open environments (forest and shrub cover = cover environments; improved pasture and prairie = open environments). Distance measured in 10 m intervals (10 m into cover, the edge, and 10 to ≥60 m into open environment).	Panthers use edge as a hunting environment so the probability of presence will be higher close to edge environments [13].
Forest edge density (km/4.5 km^2^)	Forest edge defined as the line between forest polygons (upland and wetland forests) and any land cover polygon forming a natural edge with the forest (excludes urban, crops, mines). Forest edge density measured within 4.5km^2^.*	Surrogate for prey abundance / availability as primary prey species (white tailed deer and hog) are considered edge species [14,15]. Probability of presence will be higher in areas of high forest edge density
Forest patch size (ha)	Patch size (ha) of wetland and upland forests	Panthers select for the smallest (0.1–1.0 ha), intermediate (5.1–10.0 ha) and largest (>1000 ha) classes of forest patch size [12]. Probability of presence will be higher in these patch sizes.
Percent forest cover	The percent of upland and wetland forests within 4.5km^2^. *	Panthers select for upland and wetland forests and panthers use upland forests more than other habitat classes during nighttime hours [12]: Probability of presence will be greater in areas of high % forest cover.
Improved pasture patch size	Patch size (ha) of improved pastures.	Small patches of improved pasture that lie within a heterogeneous landscape create hunting edge for panthers and will have higher probability of presence [12,13]. Large patches create areas of poor stalking habitat that will have lower probability of presence.
Land cover	Land cover classes reclassified from the FNAI Cooperative Land Cover database v.2.3 (upland forest, wetland forest, shrub-brush-prairie, non-forested wetlands, unimproved pasture, improved pasture, row crops, citrus groves).	Panthers select for upland and wetland forest [12]. Probability of presence will be highest in these land cover classes.
Dominant land cover	Land cover class that occurs most often within 4.5 km^2^.*	Panthers select for upland and wetland forest [12]. Probability of presence will be highest in areas where these land cover classes occur most often.

*Scale based on average area used by panthers during a 24-hr period.

We evaluated model performance using the Area Under the Curve (AUC) of the Receiver Operating Characteristic (ROC) curve, which is a plot of sensitivity and specificity, with sensitivity evaluating how well data correctly predicts presence, and specificity a measure of correctly predicted absences [29]. The AUC value provides a single measure of model performance and indicates the probability that the model will rank a randomly chosen presence point higher than a randomly chosen absence (in our case random) point [30]. AUC values close to 0.5 indicate that the fit is no better than that expected by random, and values of 1.0 indicate a perfect fit [31]. We chose cross validation as a sampling technique to obtain training and testing points because it is considered the best approach if you are using pseudo-absences (random background points) instead of absence data [32].

To test the significance of the model we compared our AUC value against a null distribution of expected AUC values based on random points because the literature suggests that AUC values can give a misleading evaluation of model performance [33,34,35,36]. We used random rather than actual calf depredation locations in testing our model to avoid bias because 17 of 27 (63%) verified depredation locations documented during this study and by FWC responses to rancher complaints occurred on a single ranch and the majority (59%) of those locations were from a single pasture. We generated our null model following the methods of Raes and ter Steege [36]. To do so, we created 1,000 random points within the same area used to generate our panther presence model. We used 1,000 random points because we used 1,000 panther presence locations in our model and repeated this process 1,000 times, therefore creating 1,000 different sets of 1,000 random points each. We then ran these points through MaxEnt using the same environmental variables used in the panther presence model. MaxEnt output consisted of 1,000 AUC values, each representing a different set of 1,000 random points. We compared the value of our panther presence model to the values of the null model to assess whether it fell into the top 5%, which would indicate that our model performed better than random at P = 0.05.

To assess whether the panther hunting habitat model could identify depredation sites as risky habitat we examined the mean probability of presence around 28 documented calf depredation (cache site) locations identified during our study and by the FWC during 2010–2014 [19] (S2 Table). In each case, we assumed the kill took place within 100 m of each cache site [22], created circular buffers (radius = 100 m) around each cache site, and used the panther hunting habitat model to calculate the mean probability of panther presence within this area. We hypothesized that untagged calves found by ranchers would be in more open areas with a lower probability of panther presence and used a two-sample t-test (SPSS 22, IBM, New York, NY, USA) to compare the probability of panther presence between tagged and untagged calf depredation locations. We used a Mann-Whitney U-test (SPSS 22, IBM, New York, NY, USA) to compare the probability of panther presence between the study areas and also calculated the percent of each study area that contained greater than 50% probability of panther presence.

## Results

### Calf Depredation

The degree of calf depredation by panthers differed between JB Ranch and IM Ranch. On JB Ranch, panthers killed four tagged calves during the first study season and six in the second study season (Table 2). This represented an average tagged calf loss to panthers of 5.3%/yr and accounted for 53% of total calf mortality and 90% of all predation events in the study herd over the two years on JB Ranch. On IM Ranch, one tagged calf was killed by a panther in the first study season and no tagged calves were confirmed as killed by panthers in the second study season (Table 2). This represented an average calf loss to panthers of 0.5%/yr and accounted for 12.5% of total calf mortality and 33% of all predation events in the tagged study herds over the two years on IM Ranch.

**Table 2:** Total number of calf mortalities, causes of death, and average values (%) documented for radio-tagged domestic calves on the JB Ranch and IM Ranch study areas in southwest Florida over two study seasons during September-April 2011–12 and 2012–13.

			Cause of Death			
Study Site	Number of Tagged Calves	Total and % Calf Mortality	Florida Panther	Black Bear	Unknown Predator	Non-Predation
JB	190	19 (10%)	10 (5.3%)	1 (0.5%)	0 (0.0%)	8 (4.2%)
IM	219	8 (3.7%)	1 (0.5%)	1 (0.5%)	1 (0.5%)	5 (2.3%)

Calf loss to panthers over both years was significantly higher (X^2^_1_ = 8.981, *p* = 0.003) on JB Ranch than IM Ranch. However, it is important to note that tagged calves on the IM Ranch were intermingled with calves without ear-tag transmitters, which spread the risk of predation among tagged and untagged individuals and may have influenced depredation rates in the study herd. In addition to panther depredations, both ranches lost one tagged calf (0.5%/yr) over the two-year study to black bears.

Predators killed calves of different ages and weights (S3 and S4 Tables). Panthers killed tagged calves ranging from four days old and 16 kg, to 72 days old and 82 kg (avg. = 43 kg ± 6 kg). Untagged calves killed by panthers ranged from 10 days old and 36 kg to 255 days old and 160 kg (avg. = 95 kg ± 15 kg). Combining tagged and untagged calves, the average weight of depredated calves was 63 kg ± 9 kg. The average age of tagged calves killed by panthers was 27.2 ± 7.0 days (range 4–72). Tagged calves killed by panthers were significantly younger (*z* = -2.293, *p* = 0.023) than the average age of all available calves (68.2 ± 2.2 days; range 1–199) in the study herds. The majority of calves (100% of tagged and 88% of total) killed by panthers were between 0–3 months old. The majority (82%) of all panther depredations on JB Ranch occurred between September and February, which corresponds to the calving season on that ranch.

Florida panthers of different sexes and age classes killed calves. On JB Ranch, one adult male panther, identified by a notched ear, killed three tagged calves and one untagged calf over the two study seasons. Several calves were killed by young males, which may have been the same or separate individuals. An adult female accompanied by a juvenile female also killed a calf. On IM Ranch, an adult male killed one calf.

Calf losses on the two ranches also occurred due to causes other than predators, such as health issues and abandonment (Table 2). Across both ranches, all non-predation calf mortalities except for one occurred when calves were <10 days old. Total tagged calf loss from all sources was 10%/yr on JB Ranch and 3.7%/yr on IM Ranch, and was significantly higher (X^2^_1_ = 6.647, *p* = 0.010) on JB Ranch. However, MARK survival analysis, which takes into account the number of days the calves were monitored on each ranch, indicated that total calf survival rates did not differ between ranches (model 6) or between years (model 9) (Table 3). The top MARK model indicated calf study herd survival rates (S ± SE) were lowest during both seasons on the JB Ranch and the first season on the IM Ranch and these survival rates (0.996 ± 0.789 E -003) did not differ statistically. Calf survival rates (0.999 ± 0.614 E-003) were significantly different and higher for the IM Ranch study herd in the second study season.

**Table 3:** Results of MARK survival analysis using 9 candidate models to test differences in domestic calf survival on JB Ranch and IM Ranch with model selection based on corrected Akaike Information Criterion (AIC_c_).

	Model	AICc	AICc Weight	Num. Par	Deviance
1	JB2 = JB1 = IM1 v IM2	335.2245	0.30038	2	90.2869
2	JB1 = IM1 v JB2 v IM2	335.6019	0.24872	3	88.6627
3	JB1 = JB2 v IM1 v IM2	337.0818	0.11867	3	90.1426
4	JB1 v JB2 v IM1 v IM2	337.4871	0.09691	4	88.546
5	JB1 = IM1 = IM2 v JB2	337.5710	0.09292	2	92.6334
6	JB1 = JB2 v IM1 = IM2	338.9277	0.04715	2	93.9901
7	JB1 = JB2 = IM1 = IM2	339.1859	0.04144	1	96.2493
8	IM1 = IM2 v JB1 v JB2	339.3326	0.03851	3	92.3934
9	JB1 = IM1 v JB2 = IM2	341.1803	0.01529	2	96.2427

Variables in model are noted by site/year. For example, JB2 represents JB Ranch calf survival in study year 2 and IM1 represents IM ranch calf survival in study year 1.

“V” indicates a difference in survival rate and “=“ indicates calf survival is equivalent.

### Panther Hunting Habitat Model and Predation Risk

**Fig 2:**
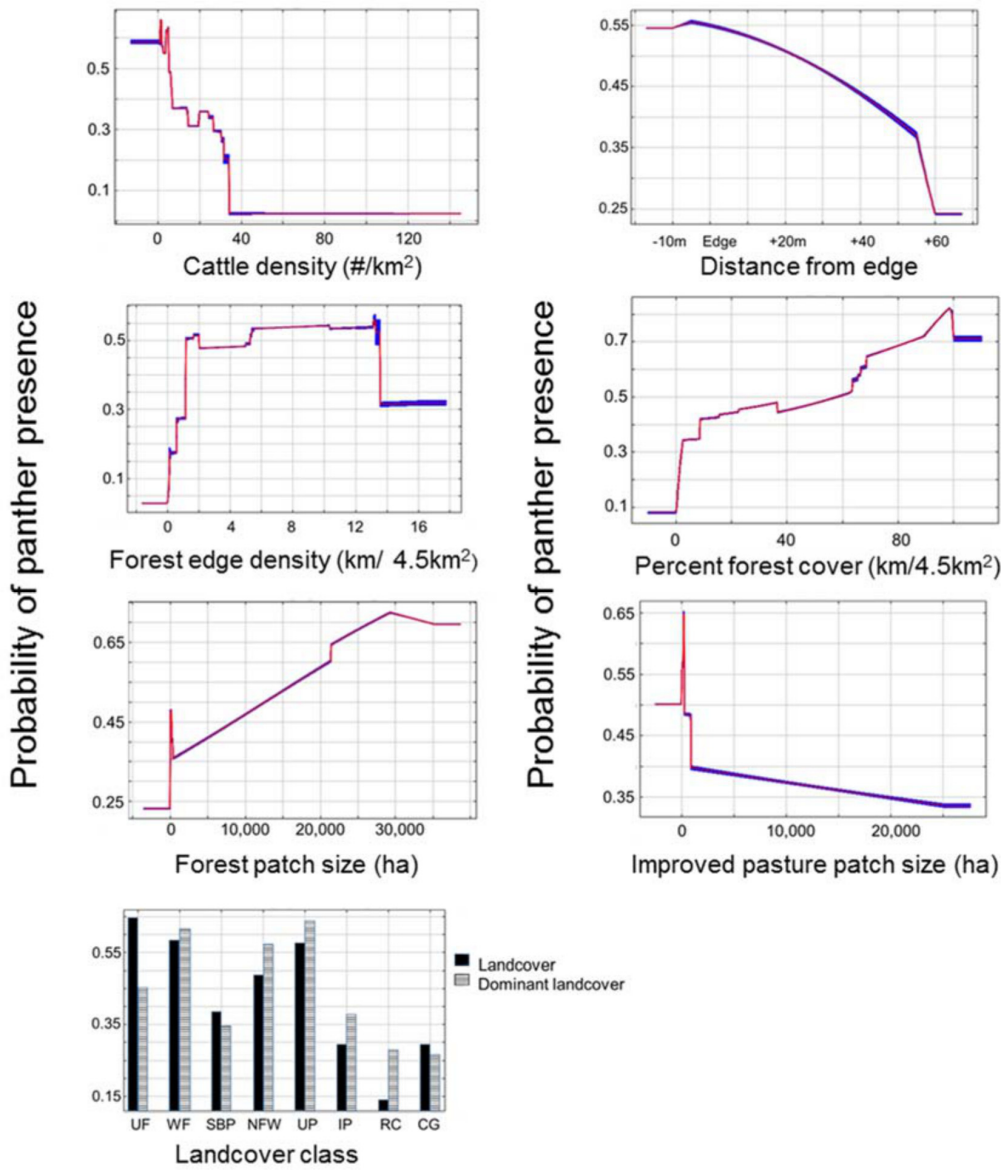
Probability of panther presence (y-axis) associated with changes in environmental variables (x-axis) as predicted by the panther hunting habitat model. UF = Upland Forest, WF = Wetland Forest, SBP = Shrub-Brush-Prairie, NFW = Non-Forested Wetland, UP = Unimproved pasture, IP = Improved Pasture, RC = Row Crops, CG = Citrus Groves.

The panther hunting habitat model produced an AUC value of 0.778, which is considered moderate performance [37]. The model was highly significant in that it predicted documented panther locations statistically better (*p* < 0.001) than 1,000 random models, which means that its accuracy was significantly higher than what would be expected by chance alone. Environmental variables contributed to the model in different ways (Table 4) and positive influences included the size of forest patches and percentage of forest cover (Fig 2). Forest edge density also had a positive influence until edge densities approached 3,000 m/km^2^ (13 km/4.5 km^2^), after which the probability of panther presence began to decline (Fig 2). Cover types that had the highest probability of panther presence included upland forest, wetland forest, and unimproved pasture (Fig 2).

**Table 4:** Percent contribution of the environmental variables to the panther hunting habitat model.

Variable	% Contribution
Cattle density	23.2
Forest patch size (ha)	19.5
Distance from edge (m)	16.4
Land cover	11.8
Percent forest cover	9.1
Forest edge density (km/4.5 km^2^)	8.4
Dominant land cover	8.4
Improved pasture patch size (ha)	3.0

Negative influences on the probability of panther presence included cattle density, increasing size of improved pasture, and distance from edge habitat (Fig 2). The probability of panther presence also decreases as the distance from edge (from cover) increases, and the probability of presence decreases substantially at distances of ~55 m outside of a cover environment.

**Fig 3:**
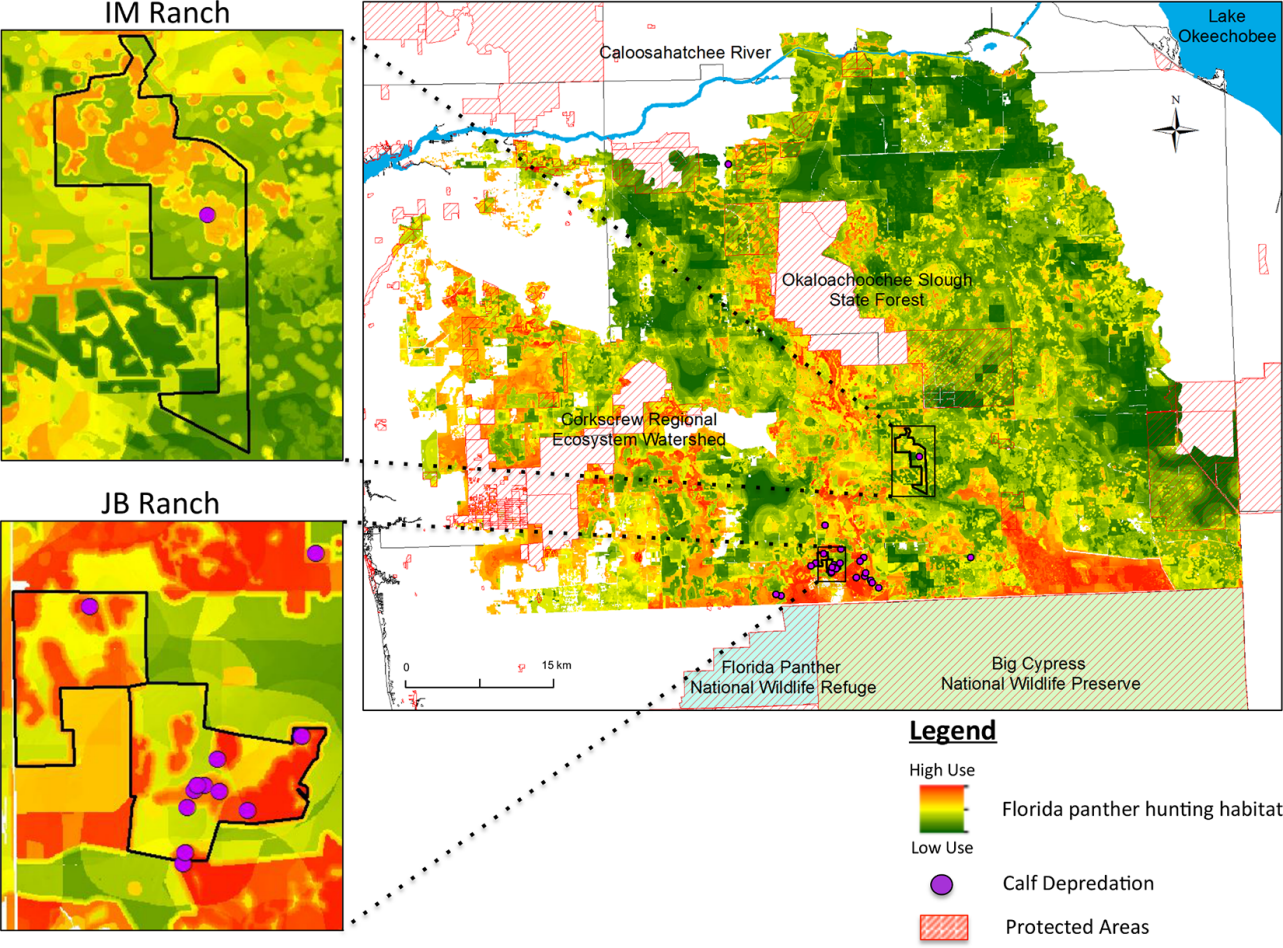
Florida panther hunting habitat model created with MaxEnt, showing the probability of panther presence at night on private lands within the primary, secondary, and dispersal habitat zones in southwest Florida. Inset maps display JB Ranch and IM Ranch study areas with calf depredation sites (ranch maps not to scale). Corkscrew Regional Ecosystem Watershed and Okaloachoochee Slough State Forest were not included in analysis because landcover differs substantially from private lands.

**Fig 4:**
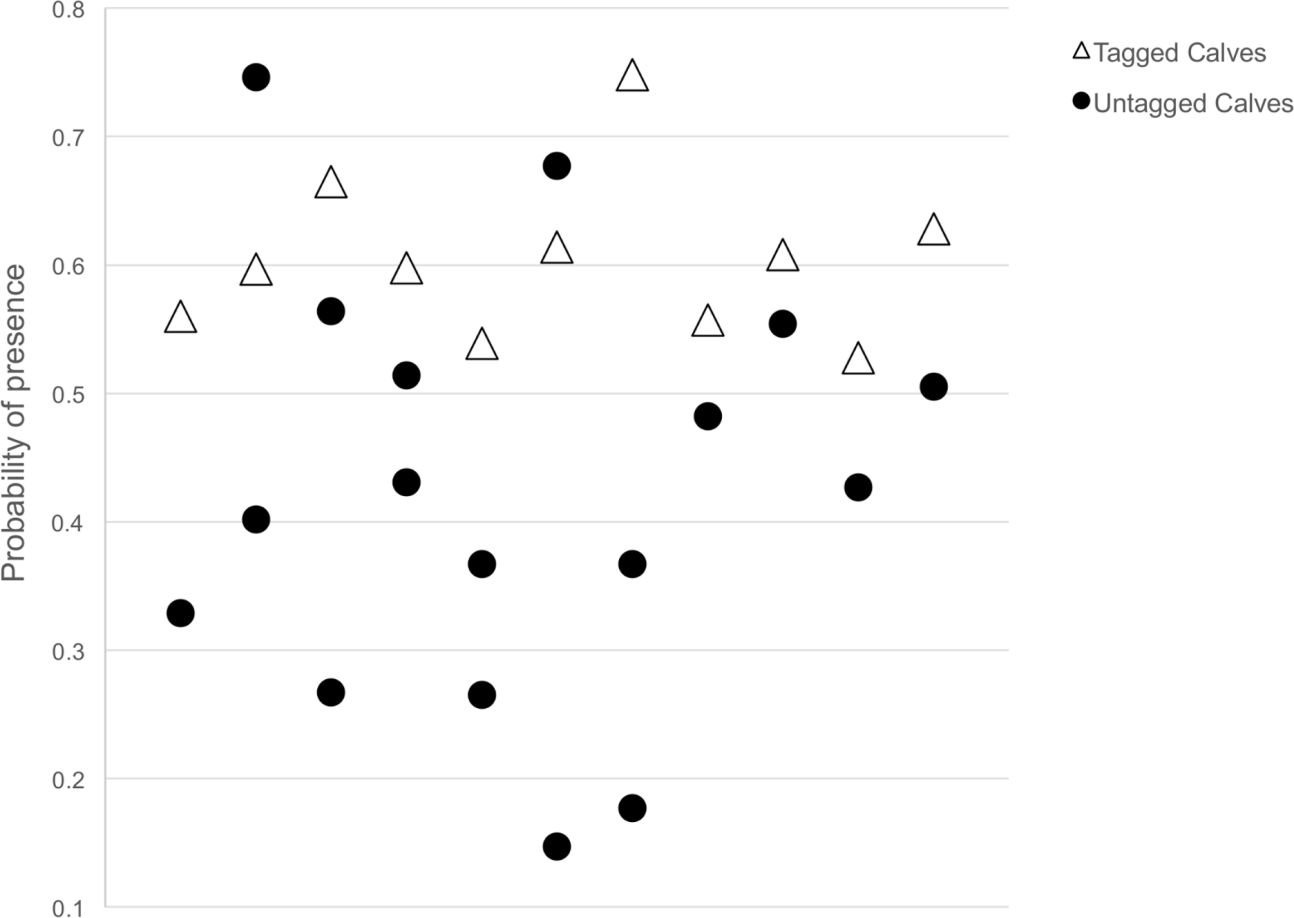
Probability of panther presence predicted by the panther hunting habitat model at locations of tagged and untagged calf depredations documented in southwest Florida. Points are arbitrarily distributed along the x-axis for illustrative purposes (to prevent overlapping points) and do not reflect a time series.

The panther hunting habitat model indicated that the JB Ranch study area contained a higher probability of panther presence (0.50 ± 0.16) than the IM Ranch study area (0.29 ± 0.14), and that these differences were significant (*U* = 463,780,767; *p* < 0.05) (Fig 3). To place this in perspective, nearly half (47%) of the JB Ranch study area had a probability of >0.50 of panther presence, whereas only 3% of the IM Ranch study area had a probability of >0.50 of panther presence. Model results were consistent with analysis comparing the amount and distribution of land cover types across each ranch [38] (S5 Table). We found that the mean probability of panther presence associated with the kill site (100 m radius around cache site) differed between tagged and untagged calves. The probability of panther presence was 0.60 around tagged calves (*n* = 11), 0.42 around untagged locations (*n* = 17), and 0.50 around all locations (*n* = 28) (Fig 4). Depredation sites of tagged calves (0.60 ± 0.06) had a significantly greater (t_22_ = 4.08, *p* < 0.05) probability of panther presence than depredation sites of untagged calves (0.42 ± 0.16).

## Discussion

### Calf Depredation

Calf depredation events were not evenly distributed throughout the year, among calf age and weight classes, or between ranches. The majority (82%) of all calf depredation events (tagged and untagged) documented on JB Ranch occurred during the calving season (September-February). These results are consistent with JB Ranch records that indicate calves are rarely lost to any causes between April and July (R. Priddy, JB Ranch, personal communication). While panthers killed calves as large as 160 kg, panthers selected for smaller calves and the majority (88%) of calves killed were <90 kg. This approximates the average size of adult feral hogs (<90 kg) and white-tailed deer (43–57 kg) in south Florida, which are reported to be the preferred prey of the Florida panther [39,40,41]. Finally, depredation rates varied between the two ranches and may have been influenced by the amount of panther hunting habitat as our model indicated that JB Ranch provided a more optimal hunting environment for panthers, which was consistent with the higher rates of calf depredation documented on the JB Ranch study area.

The differences we documented in depredation rates between ranches may also have been influenced by differences in the percentage of tagged calves and the age distributions of tagged calves in the two study herds. Whereas all (100%) of the calves were tagged within the JB Ranch study area, only 30% of the calves were tagged within the IM Ranch study area. This is because the IM Ranch herd was much larger and budgetary limitations restricted the number of calves that could be tagged. Additionally, because we tagged the first 100 calves born in the IM Ranch study herd, untagged calves born later would have been smaller and more vulnerable as suggested by our depredation data. Consequently, the possibility exists that panthers killed untagged calves on the IM Ranch study area that went undocumented. In addition to having better coverage with radio-tags on JB, during both study years the calves on JB ranch were monitored for a longer period of time (31 weeks vs. ≤23 weeks), which gave us more opportunity to document depredation events. Due to these factors, it is possible that we underestimated the actual depredation rates that occurred within the combined herd of tagged and untagged calves on IM Ranch.

The difference in monitoring periods between the ranches may have also influenced the results of the MARK survival analysis. While the proportion of radio-tagged calves that died from all causes was almost three times as high on JB (10%) than IM (3.7%), survival analysis indicated that the rates of mortality were not significantly different between ranches. Calf mortality is a function of the rate of mortality and the duration of exposure to mortality agents [42] which suggests that the difference in total mortality observed between JB and IM may have been influenced by the difference in the number of weeks we monitored the herds on each ranch (duration of exposure). Calf loss reports from JB and IM support this idea. During the two study years, average total calf loss from all causes was 19%/yr for the JB Ranch study herd (C. Jacobs, University of Florida IFAS, unpublished data) and 15.75%/yr in the IM Ranch herd of tagged and untagged calves, (B. Stoner, IM Ranch, personal communication) indicating that total calf mortality was not very different between the ranches.

### Florida Panther Hunting Habitat Model

Results from the model suggested that panthers are most likely to use landscapes with low cattle densities, large forest patches, a high percentage of forest cover, small patches of improved pasture, and areas of upland forest (Fig 2). Panthers were also more likely to use upland forests than other land cover classes, despite the fact that upland forests were not the dominant land cover (Fig 2). This suggests that panthers select for upland forests at night, which is consistent with data reported by Onorato et al. [12]. The negative influences of cattle densities, increasing size of improved pasture, and distance from edge are all related because higher cattle densities require larger improved pastures, which contain less optimal hunting habitat as the distance from edge habitat into improved pasture increases [13]. Conversely, small open patches near edge environments provide good hunting habitat, which may explain why small patches of improved pasture have a high probability of panther presence (Fig 2). Although improved pastures are not traditionally viewed as ideal panther habitat, our results suggest that small patches of improved pasture interspersed with other cover types can provide high quality panther habitat. Finally, the model indicated that panther use of open habitats declines rapidly as the distance from edge increases.

Calculations from model results indicated that the mean probability of panther presence was significantly higher around depredation sites of tagged calves than untagged calves. This difference was likely influenced by the fact that the locations of untagged calf depredations were biased towards more open areas where ranchers could find depredated carcasses, with 65% of the untagged panther depredations occurring in areas identified as open, low use environments (Fig 4).

The fact that most untagged calves killed by panthers and found by ranchers were found in open areas has important implications. The first is that untagged depredated calves are unlikely to be found unless they are killed and left in relatively open areas. Typically, pumas do not leave prey in the open and instead conceal their prey under brush and in dense cover to hide the carcass from scavengers [22]. Because all tagged calves killed by panthers were cached in areas predicted by the model to contain higher-risk, this suggests that there may be more calves killed in high risk environments than are being found and reported by ranchers. For example, while total calf loss of tagged calves was 10% on JB Ranch, the average total calf loss from this herd over the two study years was reported at 19%/yr. The 9% difference may be due to failed pregnancies and potentially from calves that were born but never found and tagged, some of which may have been killed by panthers.

Additional implications regarding depredated calves found in open areas is that calves may be opportunistically killed by panthers traveling through pastures and that panthers may learn to hunt calves in open pastures. Although Florida panthers are reported to use edge and forested environments substantially more than open areas [12], pumas from other regions are reported to use a wide range of both open and cover environments [43], especially in regions that support high prey biomass and lack large terrestrial competitors such as wolves (*Canis lupus*) [44]. Learned behavior among predators is well documented and is a function of exposure to the prey species and practice [45] and learned hunting behaviors have been documented for large felids with wild prey and livestock [44,46,47]. If some panthers are targeting calves as prey, livestock depredation may be correlated with the calving season, which is consistent with our data and has also been reported for jaguars and pumas in Venezuela [48].

Although the panther hunting habitat model could not predict the exact location of every calf depredation site, tagged depredations occurred in areas where the model predicted a higher probability of panther presence. The panther hunting habitat model, therefore, provides a means for evaluating the quality of panther hunting habitat and the corresponding risk of depredation to livestock across the landscape. While predation risk models can provide similar results, it may be difficult to obtain a sufficient number of livestock depredation locations to create these models, which typically use >50 data points [49,50]. Additionally, data points used in predation risk models may be more evenly distributed across a study area compared to livestock depredations that may be confined to a pasture or ranch. Consequently, a habitat suitability model such as the panther hunting habitat model may be a useful alternative to predict risky habitat, especially if the model focuses on the hunting environment. Similar conclusions were reached by Abade et al. [51] in their assessment of livestock depredation risk in Tanzania’s Ruaha National Park.

### Potential Utility of Depredation Risk Models

Depredation risk models have potential practical applications for both livestock husbandry and large carnivore conservation. For example, they could be used to inform livestock management decisions such as the placement of calving areas. And, in regions where livestock management is not a practical solution to reducing depredation and where funds for compensation are available, they could be used to prioritize ranches for participation in programs based on quality of predator habitat and corresponding risk of depredation to livestock.

Livestock management techniques recommended to reduce the availability and vulnerability of calves to predators, such as shortening the calving season and implementing intensive management practices [42,52], may not be practical in some areas. For example, most large cattle operations in Florida aim for a 90–150 day calving season, the length of which is dictated by the extensive management system and cattle breeds that tend to have a less synchronized breeding season than breeds adapted to northern climates (G. C. Lamb, University of Florida IFAS, personal communication). Implementing intensive management practices such as maintaining high stocking rates and moving cattle around the landscape can decrease the availability and vulnerability of calves by reducing encounter rates and disrupting a predator’s ability to learn the location of available prey, and by enhancing group anti-predator strategies such as improved vigilance, predator confusion, and communal defense (T. Kaminski, Mountain Livestock Cooperative, personal communication). And anti-predator techniques such as the use of livestock guarding dogs can be effective, but only when cattle are not widely dispersed [53].

Due to the lower quality of forage and economic limitations, the majority of large Florida ranches are extensively managed, which means that cows range over large pastures and are not closely monitored. This allows cows to become widely dispersed and increases both their availability and vulnerability to panthers. Implementing intensive management practices on large Florida cattle ranches would increase the logistical and financial challenges of commercial cattle production and would require more fencing, labor, and the creation of larger improved pastures to support higher densities of cattle (C.W. Stoner, IM Ranch, personal communication). Whether these intensive management practices are a viable economic option for some large ranches in south Florida is beyond the scope of this study, but converting unimproved pasture to improved pasture to implement anti-predator management strategies would eliminate important habitat for panthers and their prey and would be detrimental to panther conservation and recovery efforts.

Because it may not be feasible for ranches with landscapes favorable to panther recovery to implement livestock management practices that reduce depredation risk, a compensation program is likely needed to offset the cost of calf loss to panthers and to incentivize landowners to maintain panther habitat. Designing effective compensation programs for livestock depredation is challenging [54] and many programs require documentation of depredations, such as the U.S. Department of Agriculture’s Livestock Indemnity Program [55]. Although calf depredation by panthers would qualify for this program, it may be problematic in Florida where livestock management practices, the landscape, and the behavior of panthers make it difficult to verify depredation events. Programs that do not involve verifying kills are more applicable to Florida, such as those that use a Payment for Ecosystem Services (PES) strategy. In a PES program, the landowner is compensated based on some performance criteria related to conservation goals, such as the number of young produced, prey density, or the conservation and management of desired habitat [2]. A conservation incentives program using a PES design may provide a mechanism for compensating cattle operations for real or potential calf losses because it would not require verification of depredations and instead could be based on actions that contribute to conservation goals, such as the amount of panther habitat maintained and managed on a ranch.

## Conclusions

Panther conservation and recovery relies on the preservation of large parcels of suitable habitat, but increased risk of calf depredation from panthers may prove to be a disincentive for some ranch owners to maintain these landscapes. It is therefore important to reduce the economic impact of calf depredation through proactive management techniques or compensation and promote the conservation of panther habitat on ranches through incentive-based programs. Implementing intensive livestock management practices to reduce depredation is not considered a practical option for large cattle ranches in Florida, so compensation strategies are needed that are both equitable and that promote continued conservation of important panther habitat. Our panther hunting habitat model provides two important measures; it quantifies high quality panther habitat and provides a measure of depredation risk to calves. There are various ways that this information could be incorporated into a PES program that also compensates for livestock loss. For one, payments could be scaled based on the amount of high quality/risky habitat on a ranch. This would be an ideal measure because it covers both the performance related to conserving habitat as well as the potential cost of having predators on the landscape. In other words, it rewards ranchers for managing high quality habitat while compensating them for the risk associated with maintaining that habitat. Additionally, the amount and quality of hunting habitat could be used to prioritize or categorize ranches for participation in compensation programs [50, 51,56,57,58]. Following the recommendations based on this research, the USFWS is planning to use the hunting habitat model we developed in a proposed PES program to compensate landowners for livestock loss and incentivize the conservation of panther habitat on private lands (E. Myers, U.S. Fish & Wildlife Service, personal communication). Because it is anticipated that the PES program will have limited funding, the model will be used to help identify those ranch landscapes most important to Florida panther recovery.

If large carnivore conservation and recovery is dependent on maintaining suitable habitat on private lands, strategies designed to compensate and incentivize landowners for managing large carnivore habitat will promote conservation efforts. The panther hunting habitat model presented here represents an approach that may be useful for addressing livestock depredation conflicts for other carnivores and areas worldwide by providing a means to prioritize and categorize private lands for participation in a PES program that incentivizes the conservation of large carnivore habitat and compensates landowners for the associated risks to livestock.

## Supporting Information

S1 Dataset Florida panther GPS locations used in MaxEnt to create hunting habitat model. (XLSX)

S1 Fig Map illustrating the distribution of Florida panther GPS locations used to create panther hunting habitat model. (TIFF)

S1 Table Landcover reclassification for MaxEnt Analysis on private lands within the Florida panther primary zone, excluding landcover classes that represented less than 1% of the study area. (DOCX)

S2 Table Data associated with depredations used to compare the probability of panther presence between tagged and untagged depredation locations. (DOCX)

S3 Table Calf and predator information documented for calf depredations (both tagged and untagged calves) that occurred on the JB Ranch during September 2011-April 2013. (DOCX)

S4 Table Calf and predator information documented for calf depredations (both tagged and untagged calves) that occurred on the IM Ranch during September 2011-March 2013. (DOCX)

S5 Table Comparison of landscape variables between the JB Ranch and IM Ranch at three scales. The Study Area scale refers to the rangeland used by the two study herds (JB = 268 ha; IM = 913 ha). The 5- and 10-km Buffer Zones refer to the size of the radii of a circle (buffer zone) surrounding each study area. Landscape variable metrics include percent cover and mean patch size (MPS). Poor Stalking Habitat was created as a separate variable and defined as improved pasture >90 m from the edge of forest or other cover. Upland forest patch density quantifies the number of upland forest patches >0.5 ha/100 ha, and upland forest patch connectivity provides the average (mean) distance between the center of forest patches in each study area.
(DOCX)
