# Supplementary figures and images for: A Conservation-Based Approach to Compensation for Livestock Depredation: The Florida Panther Case Study
Source: PLoS One. 2015 Sep 30;10(9):e0139203. doi: 10.1371/journal.pone.0139203 (PMC4589380; doi:10.1371/journal.pone.0139203)

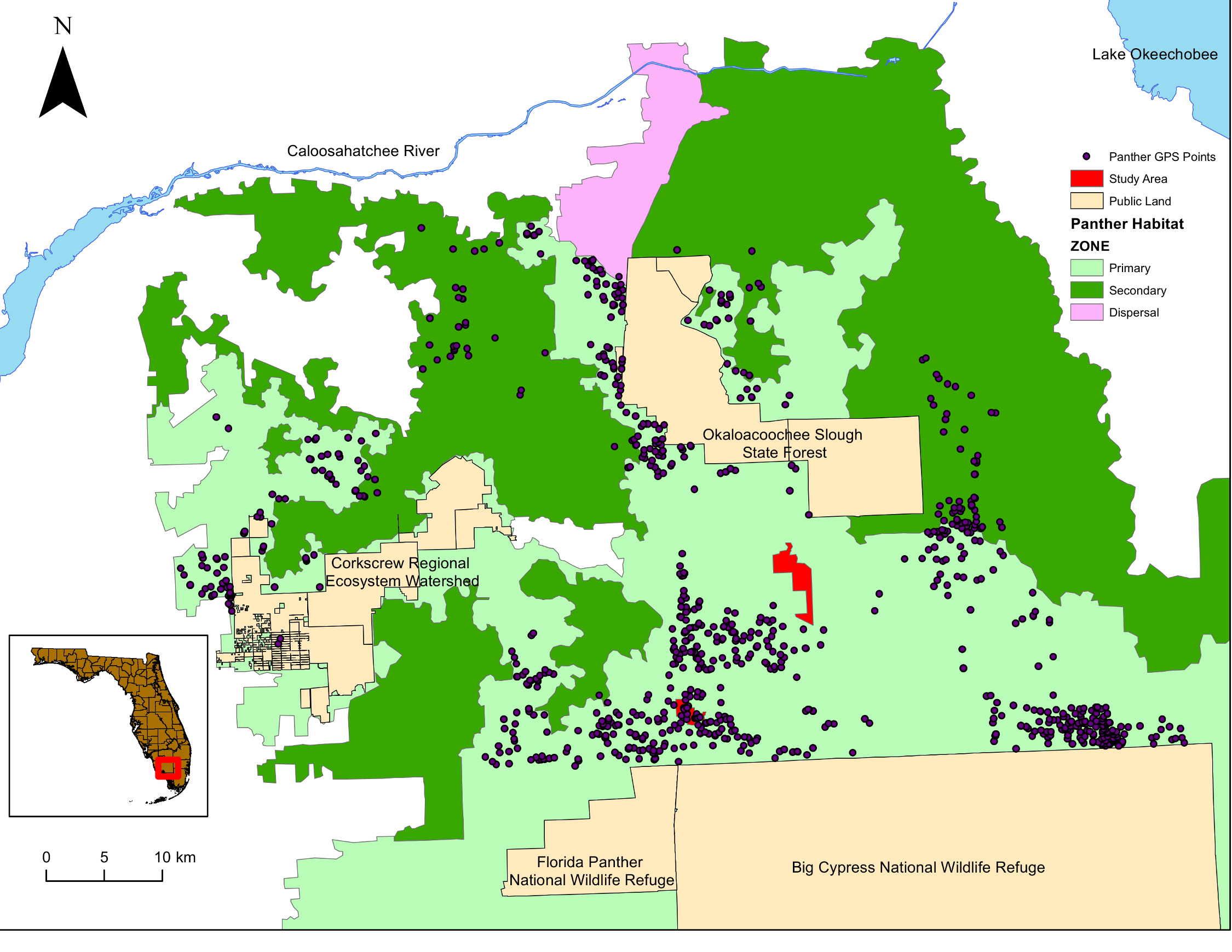

Supplement: S1 Fig — (TIFF) [file pone.0139203.s002.tiff]
