# Supplementary material for: A Conservation-Based Approach to Compensation for Livestock Depredation: The Florida Panther Case Study
Source: PLoS One. 2015 Sep 30;10(9):e0139203. doi: 10.1371/journal.pone.0139203 (PMC4589380; doi:10.1371/journal.pone.0139203)
Supplement: S1 Table — (DOCX) [file pone.0139203.s003.docx]

**S1 Table. Landcover reclassification for MaxEnt Analysis on private lands within the Florida panther primary zone, excluding landcover classes that represented less than 1% of the study area.**

| Reclassified Landcover Class | Original Landcover Class  (CLC v. 2.3) | Cover / Open | Forest / Non-Forest |
| --- | --- | --- | --- |
| Upland Forest | Hardwood Forested Uplands | Cover | F |
|  | Rockland Hammock | Cover | F |
|  | Other High Pine | Cover | F |
|  | Sandhill | Cover | F |
|  | Mesic Flatwoods | Cover | F |
|  | Mixed Hardwood Coniferous | Cover | F |
|  | Maritime Hammock | Cover | F |
|  | Tree Plantations | Cover | F |
|  | Exotic Plants - Australian Pine | Cover | F |
|  | Exotic Plants - Melaleuca | Cover | F |
|  | Rural Lands - Rural Open Forested | Cover | F |
|  | Rural Lands - Oak-Cabbage Palm Forests | Cover | F |
|  | Rural Lands - Rural Open Pine | Cover | F |
| Wetland Forest | Cypress / Tupleo | Cover | F |
|  | Strand Swamp | Cover | F |
|  | Other Coniferous Wetland | Cover | F |
|  | Wet Flatwoods | Cover | F |
|  | Mixed Wetland Hardwoods | Cover | F |
|  | Hydric Hammock | Cover | F |
|  | Other Wetland Forested Mixed | Cover | F |
|  | Wet Coniferous Plantations | Cover | F |
|  | Exotic Plants - Exotic Wetland Hardwoods | Cover | F |
| Non-Forested Wetlands | Freshwater Non-forested Wetlands | Open | NF |
|  | Prairies and Bogs | Open | NF |
|  | Wet Prairie | Open | NF |
|  | Freshwater Marshes | Open | NF |
|  | Floodplain Marsh | Open | NF |
|  | Glades Marsh | Open | NF |
|  | Non-vegetated Wetlands | Open | NF |
| Shrub-Brush-Prairie | Scrub | Cover | NF |
|  | Scrubby Flatwoods | Cover | NF |
|  | Dry Prairie | Open | NF |
|  | Shrub and Brushland | Cover | NF |
|  | Exotic Plants - Brazilian Pepper | Cover | NF |
|  | Rural Lands - Rural Open | Cover | NF |
| Unimproved Pasture | Unimproved Pasture | Cover | NF |
| Improved Pasture | Improved Pasture | Open | NF |
|  | Agriculture - Cropland / Pasture | Open | NF |
| Row Crops | Agriculture | Open | NF |
|  | Agriculture - Row Crops | Open | NF |
|  | Agriculture - Field Crops | Open | NF |
|  | Agriculture - Sugarcane | Open | NF |
|  | Agriculture - Fallow Cropland | Open | NF |
|  | Agriculture - Vineyard & Nurseries | Open | NF |

**Table A1. Continued**

| Reclassified Landcover Class | Original Landcover Class  (CLC v. 2.3) | Cover / Open | Forest / Non-Forest |
| --- | --- | --- | --- |
| Row Crops | Agriculture - Vineyard & Nurseries | Open | NF |
|  | Agriculture - Tree Nurseries | Open | NF |
|  | Agriculture - Sod Farms | Open | NF |
|  | Agriculture - Ornamentals | Open | NF |
|  | Agriculture - Feeding Operations | Open | NF |
|  | Agriculture - Specialty Farms | Open | NF |
| Citrus groves | Agriculture - Orchards / Groves | Cover | NF |
|  | Agriculture - Citrus | Cover | NF |
|  | Agriculture - Fallow Orchards | Cover | NF |
