# Supplementary material for: A Conservation-Based Approach to Compensation for Livestock Depredation: The Florida Panther Case Study
Source: PLoS One. 2015 Sep 30;10(9):e0139203. doi: 10.1371/journal.pone.0139203 (PMC4589380; doi:10.1371/journal.pone.0139203)
Supplement: S2 Table — (DOCX) [file pone.0139203.s004.docx]

**S2 Table. Data associated with depredations used to compare the probability of panther presence between tagged and untagged depredation locations.**

| Depredation ID | UTM Easting | UTM Northing | Date | Tagged (Y/N) |
| --- | --- | --- | --- | --- |
| 1 | 465586 | 2908183 | 10/2/10 | N |
| 2 | 465054 | 2907868 | 10/15/10 | N |
| 3 | 467278 | 2907519 | 10/25/10 | N |
| 4 | 467418 | 2907701 | 10/22/11 | Y |
| 5 | 477154 | 2919462 | 11/23/11 | Y |
| 6 | 467245 | 2907054 | 12/4/11 | Y |
| 7 | 467263 | 2907144 | 12/20/11 | Y |
| 8 | 467527 | 2907916 | 1/4/12 | Y |
| 9 | 469957 | 2906475 | 6/8/12 | N |
| 10 | 472386 | 2905310 | 7/7/12 | N |
| 11 | 467546 | 2907646 | 10/1/12 | Y |
| 12 | 467774 | 2907492 | 10/4/12 | Y |
| 13 | 482453 | 2908384 | 11/19/12 | N |
| 14 | 467334 | 2907656 | 11/30/12 | Y |
| 15 | 470818 | 2908664 | 12/10/12 | N |
| 16 | 471535 | 2906134 | 12/28/12 | N |
| 17 | 466467 | 2909183 | 1/2/13 | Y |
| 18 | 470884 | 2906640 | 1/23/13 | N |
| 19 | 467358 | 2907699 | 2/4/13 | Y |
| 20 | 470388 | 2908244 | 2/18/13 | N |
| 21 | 470966 | 2906970 | 2/25/13 | N |
| 22 | 468222 | 2908106 | 4/3/13 | Y |
| 23 | 466696 | 2912273 | 5/15/13 | N |
| 24 | 461708 | 2904697 | 6/24/13 | N |
| 25 | 468339 | 2909623 | 9/28/13 | N |
| 26 | 461157 | 2904876 | 11/25/13 | N |
| 27 | 471659 | 2905880 | 3/18/14 | N |
| 28 | 457107 | 2951668 | 5/19/14 | N |
