# Supplementary material for: A Conservation-Based Approach to Compensation for Livestock Depredation: The Florida Panther Case Study
Source: PLoS One. 2015 Sep 30;10(9):e0139203. doi: 10.1371/journal.pone.0139203 (PMC4589380; doi:10.1371/journal.pone.0139203)
Supplement: S3 Table — (DOCX) [file pone.0139203.s005.docx]

**S3 Table. Calf and predator information documented for calf depredations (both tagged and untagged calves) that occurred on the JB Ranch during September 2011-April 2013.**

| Study  Season | Tagged (Y/N) | Date | Calf Age (days) | Estimated Weight  of Calf (kg) | Predator (Details) |
| --- | --- | --- | --- | --- | --- |
| 1 | Y | Oct. 22, 2011 | 20 | 27-36 | Panther (Male-sized tracks) |
| 1 | Y | Dec. 12, 2011 | 25 | 32-36 | Panther (Notched-ear male) |
| 1 | Y | Dec. 20, 2011 | 46 | 50+ | Panther (Adult & juvenile females) |
| 1 | Y | Jan. 6, 2012 | 92 | 43 | Panther (Notched-ear male) |
| 2 | Y | Sept. 30, 2012 | 5 | 23 | Panther (Young male) |
| 2 | Y | Oct. 4, 2012 | 4 | 16 | Panther (Young male) |
| 2 | Y | Nov. 30, 2012 | 32 | 41 | Panther (Young male) |
| 2 | Y | Jan 1, 2013 | 49 | 180 | Panther (Young male) |
| 2 | Y | Feb. 3, 2013 | 19 | 90 | Panther (Notched-ear male) |
| 2 | Y | April 1, 2013 | 72 | 82 | Panther |
| 2 | Y | Jan 30, 2013 | 72 | 91 | Bear |
| 1 | N | July 7, 2012 | 255 | 160 | Panther |
| 1 | N* | June 11, 2012 | 255 | 150 | Panther |
| 2 | N | Dec. 10, 2012 | 72 | 82 | Panther |
| 2 | N | Dec. 14, 2012 | 7 | 27 | Bear |
| 2 | N | Dec. 27, 2012 | 90 | 91 | Panther |
| 2 | N | Jan. 22, 2013 | 53 | 82 | Panther |
| 2 | N | Feb. 17, 2013 | <14 | 36 | Panther (Notched-ear male) |
| 2 | N | Feb. 24, 2013 | 87 | 73 | Panther |
