# Supplementary material for: A Conservation-Based Approach to Compensation for Livestock Depredation: The Florida Panther Case Study
Source: PLoS One. 2015 Sep 30;10(9):e0139203. doi: 10.1371/journal.pone.0139203 (PMC4589380; doi:10.1371/journal.pone.0139203)
Supplement: S4 Table — (DOCX) [file pone.0139203.s006.docx]

**S4 Table. Calf and predator information documented for calf depredations (both tagged and untagged calves) that occurred on the IM Ranch during September 2011-March 2013.**

| Study  Season | Tagged (Y/N) | Date | Calf Age (days) | Estimated Weight  of Calf (kg) | Predator (Details) |
| --- | --- | --- | --- | --- | --- |
| 1 | Y | Nov. 23, 2011 | 6 | 23-27 | Panther (Male) |
| 1 | Y | Jan. 30, 2012 | 80 | 88 | Bear |
| 1 | Y | Feb. 5, 2012 | 68-73 | 82 | Panther or Bear |
| 1 | N | Nov. 21, 2011 | 2 | 27 | Coyote |
| 1 | N | Nov. 25, 2011 | 2 | 27 | Coyote |
| 1 | N | Feb. 20, 2012 | < 7 | 32 | Coyote |
| 2 | N | Dec. 4, 2012 | <14 | 32-36 | Coyote |
