# Supplementary material for: A Conservation-Based Approach to Compensation for Livestock Depredation: The Florida Panther Case Study
Source: PLoS One. 2015 Sep 30;10(9):e0139203. doi: 10.1371/journal.pone.0139203 (PMC4589380; doi:10.1371/journal.pone.0139203)
Supplement: S5 Table — The Study Area scale refers to the rangeland used by the two study herds (JB = 268 ha; IM = 913 ha). The 5- and 10-km Buffer Zones refer to the size of the radii of a circle (buffer zone) surrounding each study area. Landscape variable metrics include percent cover and mean patch size (MPS). Poor Stalking Habitat was created as a separate variable and defined as improved pasture >90 m from the edge of forest or other cover. Upland forest patch density quantifies the number of upland forest patches >0.5 ha/100 ha, and upland forest patch connectivity provides the average (mean) distance between the center of forest patches in each study area. (DOCX) [file pone.0139203.s007.docx]

**S5 Table. Comparison of landscape variables between the JB Ranch and IM Ranch at three scales.** The Study Area scale refers to the rangeland used by the two study herds (JB = 268 ha; IM = 913 ha). The 5- and 10-km Buffer Zones refer to the size of the radii of a circle (buffer zone) surrounding each study area. Landscape variable metrics include percent cover and mean patch size (MPS). Poor Stalking Habitat was created as a separate variable and defined as improved pasture >90 m from the edge of forest or other cover. Upland forest patch density quantifies the number of upland forest patches >0.5 ha/100 ha, and upland forest patch connectivity provides the average (mean) distance between the center of forest patches in each study area.

| Landscape Variable | Study Area | | | | | | | | 5-km Buffer Zone | | | | | | | | 10-km Buffer Zone | | | | | | |
| --- | --- | --- | --- | --- | --- | --- | --- | --- | --- | --- | --- | --- | --- | --- | --- | --- | --- | --- | --- | --- | --- | --- | --- |
|  | JB Ranch | | | | IM Ranch | | | | JB Ranch | | | | IM Ranch | | | | JB Ranch | | | | IM Ranch | | |
| Landscape Cover and Configuration Variables | Cover (%) | MPS (ha) | | Cover (%) | | MPS (ha) | | Cover (%) | | MPS (ha) | | Cover (%) | | MPS (ha) | | Cover (%) | | MPS (ha) | | Cover (%) | | MPS (ha) | |
| Upland Forest | 20 | 6 | | 14 | | 32 | | 10 | | 21 | | 8 | | 14 | | 9 | | 19 | | 10 | | 17 | |
| Wetland Forest | 5 | 3 | | 12 | | 4 | | 32 | | 21 | | 20 | | 8 | | 34 | | 25 | | 21 | | 9 | |
| Non-Forested Wetland | 6 | 3 | | 9 | | 2 | | 13 | | 31 | | 11 | | 76 | | 10 | | 8 | | 15 | | 7 | |
| Shrub-Brush-Prairie | 4 | 5 | | 10 | | 19 | | 6.11 | | 16 | | 13 | | 33 | | 7 | | 19 | | 9 | | 26 | |
| Improved Pasture | 64 | 21 | | 55 | | 64 | | 11 | | 51 | | 21 | | 118 | | 10 | | 57 | | 25 | | 139 | |
| Agriculture | n/a | n/a | | n/a | | n/a | | 25 | | 219 | | 26 | | 113 | | 27 | | 311 | | 20 | | 155 | |
| Poor Stalking Habitat | 15 | 5 | | 29 | | 33 | | n/a | | n/a | | n/a | | n/a | | n/a | | n/a | | n/a | | n/a | |
| Edge Density (m/ha) | 61 m/ha | | 45 m/ha | | | | 29 m /ha | | | | 37 m /ha | | | | 35 m /ha | | | | 48 m/ha | | | |  |
| Upland Forest Patch Density (#/100 ha) | 3.4/100 ha | | 0.4/100 ha | | | | 0.9/100 ha | | | | 1.8/100 ha | | | | 0.6/100 ha | | | | 0.9/100 ha | | | |  |
| Mean Upland Forest Patch Connectivity (Std. Dev.) | 138 m (57) | | 1100 m (1627) | | | | n/a | | | | n/a | | | | n/a | | | | n/a | | | |  |
